# Integration of Biofunctional Molecules into 3D-Printed Polymeric Micro-/Nanostructures

**DOI:** 10.3390/polym14071327

**Published:** 2022-03-25

**Authors:** Eider Berganza, Gurunath Apte, Srivatsan K. Vasantham, Thi-Huong Nguyen, Michael Hirtz

**Affiliations:** 1 Institute of Nanotechnology (INT) and Karlsruhe Nano Micro Facility (KNMFi), Karlsruhe Institute of Technology, 76131 Karlsruhe, Germany; eider.eguiarte@kit.edu (E.B.); gurunath.apte@iba-heiligenstadt.de (G.A.); srivatsan.vasantham@kit.edu (S.K.V.); 2 Institute for Bioprocessing and Analytical Measurement Techniques (iba), 37308 Heilbad Heiligenstadt, Germany; 3 Faculty of Mathematics and Natural Sciences, Technische Universität Ilmenau, 98694 Ilmenau, Germany

**Keywords:** FluidFM, 3D printing, microstructures, nanostructures, biofunctionalization, mechanical properties, scanning probe lithography

## Abstract

Three-dimensional printing at the micro-/nanoscale represents a new challenge in research and development to achieve direct printing down to nanometre-sized objects. Here, FluidFM, a combination of microfluidics with atomic force microscopy, offers attractive options to fabricate hierarchical polymer structures at different scales. However, little is known about the effect of the substrate on the printed structures and the integration of (bio)functional groups into the polymer inks. In this study, we printed micro-/nanostructures on surfaces with different wetting properties, and integrated molecules with different functional groups (rhodamine as a fluorescent label and biotin as a binding tag for proteins) into the base polymer ink. The substrate wetting properties strongly affected the printing results, in that the lateral feature sizes increased with increasing substrate hydrophilicity. Overall, ink modification only caused minor changes in the stiffness of the printed structures. This shows the generality of the approach, as significant changes in the mechanical properties on chemical functionalization could be confounders in bioapplications. The retained functionality of the obtained structures after UV curing was demonstrated by selective binding of streptavidin to the printed structures. The ability to incorporate binding tags to achieve specific interactions between relevant proteins and the fabricated micro-/nanostructures, without compromising the mechanical properties, paves a way for numerous bio and sensing applications. Additional flexibility is obtained by tuning the substrate properties for feature size control, and the option to obtain functionalized printed structures without post-processing procedures will contribute to the development of 3D printing for biological applications, using FluidFM and similar dispensing techniques.

## 1. Introduction

Three-dimensional printing has become a versatile tool for printing biomimetic scaffolds and for other biomedical applications [1]. Sophisticated lithographically generated microstructures are, in particular, used for probing or manipulating cells at the single-cell level, for elucidating cell biology and mechanics [2,3], or influencing stem cell fate [4]. While, in particular, optical methods, such as direct laser writing (DLW) and similar techniques, have made impressive progress [5], the bioactive functionalization of such microstructures remains challenging [6].

The surrounding dynamic micro/nano environment directly influences cell behaviour. In particular, the topography, stiffness, bioactive moieties, and chemical components of the surfaces regulate the response of the cells [7,8,9]. We have previously found that nanostructures, together with surface modification, could effectively control the adhesion, spreading, and activation of human blood platelets [10,11,12]. Importantly, surfaces have been developed to mimic the structure of the extracellular matrix (ECM) [13,14]. However, these structures are almost static, and do not emulate the dynamicity and function of the ECM in vivo. To overcome this limitation, shape-memory polymers (SMPs), in the form of patterns, fibres, porous scaffolds, and microspheres, have been developed to mimic dynamic changes in the ECM structure, both in vitro and in vivo [15]. The SMPs arise from materials with unique functions that regulate cell behaviours and promote tissue growth. The adjustment of structured surfaces, as well as chemical components, can further optimize contact environments for cell response/sensing and tissue regeneration.

While methods for the fabrication of microstructures have been well established, available technology for the production of nanostructured surfaces is, to date, still limited. One recent development in the 3D printing of micro-/nanostructures is the use of nano dispensing techniques, such as FluidFM. FluidFM is based on the combination of microfluidics with atomic force microscopy (AFM), in which a hollow cantilever, with an aperture at the tip apex, can be used for precisely localized liquid dispensing [16]. Its use was quickly extended to nanolithography, e.g., for nanoparticles [17] and biomimetic membranes [18]. Depending on the size of the tip aperture, micro-/nanostructures can be printed. With this technique, hierarchical structures, at multidimensional scales, could be fabricated using the commercially available UV-curable adhesive Loctite [19,20]. This is a highly viscous ink, composed of different methacrylate esters, which are in the liquid phase and undergo cross-linking polymerization upon exposure to UV [21]. As a direct-write method, this approach is highly versatile in pattern and structure formation, and offers excellent resolution down to the tens of nanometres scale. However, little is known about the effect of substrate surface properties on the resulting printing structures, and the integration of functional groups into the printed structures to enable specific biological applications. As of now, further biofunctionalization of such structures is still lacking.

**Figure 1:**
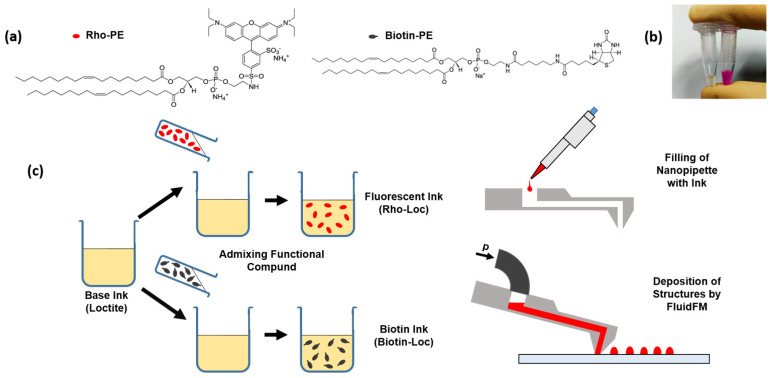
Ink preparation and printing. (**a**) Molecular structures of the phospholipids used for biofunctionalization. (**b**) Picture of the functionalized adhesive inks. (**c**) Scheme of functionalization and printing process.

Here, we demonstrate that the substrate surface properties directly affect the geometries of the printed structures. We further show the feasibility of introducing specific protein coupling sites into the printed microstructures (Figure 1). This is conducted by admixing biotin moiety-bearing amphiphilic molecules into the base adhesive ink, which are then presented on the micro-/nanostructured surfaces after curing. As no chemical post-functionalization is needed, the approach is a facile route to introduce highly specific protein binding into the polymer structures, with the possibility of selecting proteins of interest from the extensive library of biotinylated proteins, or using sandwich approaches to add non-labelled proteins via antibody capture or DNA-directed immobilization with biotinylated oligonucleotides [22,23].

## 2. Materials and Methods

### 2.1. Materials

The UV-curable adhesive Loctite 3491 (Henkel, Düsseldorf, Germany) was used as the base for all inks. Phospholipids used as functional admixtures were 1,2-dioleoyl-sn-glycero-3-phosphoethanolamine-*N*-(cap biotinyl) (biotin-PE) at a concentration of 10 mg/mL in chloroform, and 1,2-dioleoyl-sn-glycero-3-phosphoethanolamine-*N*-(lissamine rhodamine B sulfonyl) (Rho-PE) at a concentration of 1 mg/mL in chloroform, both from Avanti Polar Lipids, Alabaster, AL, USA. Functionalized inks were obtained by admixing either 10 vol% of the biotin-PE in chloroform solution (for the biofunctionalization ink) or 10 vol% of the Rho-PE in chloroform solution (for the fluorescent ink) with the base adhesive ink. A homogeneous mixture was obtained by vortexing the solution for 1–2 min.

### 2.2. FluidFM Printing

For printing of the adhesive patterns on glass or silicon substrates, the following two FluidFM systems were used: a FlexAFM (Nanosurf, Liestal, Switzerland) system and a BioAFM (JPK, Berlin, Germany) system. Experiments were performed with FluidFM nanopipettes (Cytosurge, Opfikon, Switzerland), with a nominal cantilever spring constant of 2 N/m and a 300 nm diameter nozzle/aperture at its probe end.

After the nanopipette was mounted and the reservoir was filled with 2 µL of the respective inks, 1000 mbar pressure was applied to the reservoir for 1–2 min to make the ink flow through the microchannel to the end of the probe aperture. Once the ink reached the nozzle, the ink flowed to the substrate without the need for further application of pressure. During patterning, the applied force was typically set between 10 and 20 nN. To control the feature sizes, the contact time during printing was varied between 0.5 and 5 s for nanodots whereas the writing speed was varied between 20 and 60 µm/s to print lines.

### 2.3. Characterization of Printed Structures with Atomic Force Microscopy (AFM)

The obtained structures were characterized by AFM, performed on a Dimension Icon system (Bruker, Berlin, Germany) in tapping mode. Tap300-G probes (Budget Sensors, Sofia, Bulgaria) with a resonance frequency of 330 kHz and nominal spring constant of 42 N/m were used. The indentation maps from which Young’s modulus values were extracted were obtained on a JPK BioAFM system (Bruker, Berlin, Germany), using BL-AC40TS probes with a radius of 8 nm (Asylum Research, Santa Barbara, CA, USA), with a resonance frequency of 70 Hz and nominal force constant of 2 N/m. A total of 625 force curves were analysed from areas of 2.5 × 2.5 µm^2^, with the printed feature located at the centre of the scanning area. The data obtained from the measurements were processed by fitting the force curves to the Hertz model, by selecting tip shape as pyramidical and Poisson’s ratio of 0.5. The data were processed by JPKSPM data processing software and analysed using SigmaPlot (Sysstat Software GmbH, Erkrath, Germany). Measurements were also performed over a plain glass surface to obtain control values.

### 2.4. Protein Binding

To prevent nonspecific binding, the samples were first incubated with a 10% bovine serum albumin (BSA) solution (Sigma-Aldrich, Darmstadt Germany) for 30 min at room temperature (RT). Then, biofunctionalization was demonstrated by incubating the samples with 5 µg/mL streptavidin–FITC solution (Thermo Fisher, Waltham, MA, USA) in phosphate-buffered saline (PBS) for 30 min.

### 2.5. Optical Microscopy

Optical microscopy was performed on a Nikon Eclipse Ti2 inverted fluorescence microscope (Nikon, Düsseldorf, Germany). A Texas-red filter (Nikon, Germany) was used as a light filter, and a green fluorescent protein (GFP)-compatible filter was used for visualization of biotin–streptavidin bindings.

### 2.6. Substrate Functionalization

To assess the influence of substrate wettability on the patterning feature dimensions and spreading behavior, several glass substrates were functionalized. Prior to functionalization procedures, all coverslips were sonicated in acetone, ethanol, and DI water, subsequently. Hydrophobic substrates (S1) were prepared by exposing coverslips to oxygen plasma (200 W, 50 sccm oxygen flow, in an Atto system (Diener Electronics, Ebhausen, Germany) for 5 min, and subsequently immersing them in 7-octenyltrichlorosilane (OTS) (10 vol% in toluene) for 24 h. Medium hydrophilic surfaces (S2) were used directly after cleaning, without any surface functionalization. Another type of medium hydrophilic surfaces (S3) were induced by exposing to oxygen plasma for 5 min prior to immersing in (3-glycidyloxypropyl)- trimethoxysilan (GPTMS) (2 vol% in toluene) for 4 h. Highly hydrophilic substrates (S4) were prepared by exposure to oxygen plasma for 5 min, without any further coating.

### 2.7. Characterization of Substrate Wettability

The different substrates were characterized by contact angle measurements on an OCA-20 system (DataPhysics Instruments GmbH, Filderstadt, Germany). The water contact angle (WCA) for each substrate was measured by the sessile drop method. Measurements were performed at room temperature (RT), with sample droplets of 3.0 µL volume deposited at a dosing rate of 3.0 µL/s. The contact angles were determined with the onboard software. For each substrate, 3 measurements were performed at different locations.

### 2.8. Statistical Analysis

Statistical analyses of the data were performed using SigmaPlot (version 14.0). For the printed dot features’ height measurements, 9 dots were imaged with AFM for each pulse time and on each surface. The height was then extracted in WSxM [24] from profile lines through the dots. For the printed line features’ width and height measurements, averaged profiles from the printed lines AFM images were generated using the y-average tool in WSxM, and the width and height of the averaged line profiles were measured. The obtained values for width and height were averaged from 5 printed lines for each surface. The WCAs were obtained from 3 different locations for each substrate. All error ranges given in the manuscript are the standard deviation of the respective data points, unless otherwise noted.

## 3. Results

### 3.1. Printing of Pure Adhesive

**Figure 2:**
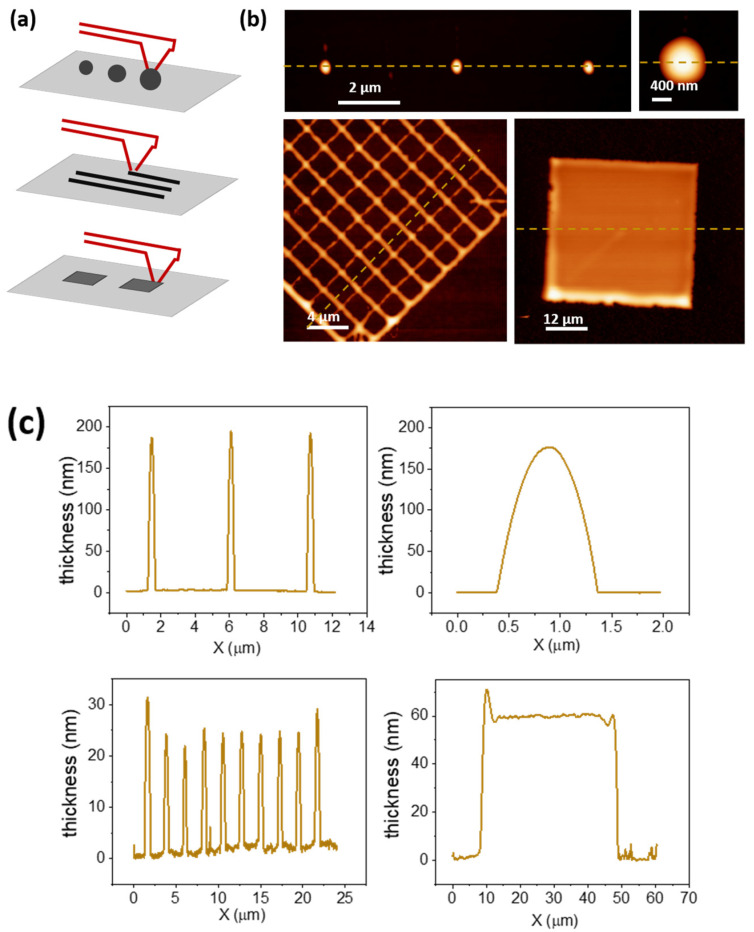
Basic geometrical patterns obtained with pure adhesive, Loctite. (**a**) Scheme of printing dots, lines, and squares as basic geometric patterns. (**b**) AFM topography images of exemplary printed structures and (**c**) corresponding profile sections of the structures at the yellow dashed line in (**b**).

Pure adhesive ink, composed of different methacrylate esters, was used to print different exemplary structures on bare glass, using a nanopipette cantilever with an aperture size of 300 nm. Different types of patterns, including dots of around 1 µm in diameter, lines, grids, and squares, can be readily printed (Figure 2 and Figure S1). Filled square patterns were obtained by drawing lines in close proximity that merged, forming structures of homogeneous thickness. It has been previously reported that, together with printing parameters such as pressure, force, and contact time, the printing direction also plays a role in the size of the printed features [19]. This effect can be observed on the grid (Figure 2b), where the line thickness varies, depending on its direction.

### 3.2. Printing with Functionalized Adhesive

#### 3.2.1. Preparation of Functionalized Adhesive Inks

Two types of modified inks, with different functional properties, were prepared for the experiments, by admixing functionalized phospholipids. For an easy assessment of miscibility, and to be able to observe the printed structure by fluorescence microscopy, a fluorescently labelled phospholipid (Rho-PE) was admixed. The resulting mixture turned out to be homogeneous upon visual inspection (Figure 1a), showing good compatibility of the solvents, which is extremely important, as the segregation of components could clog the FluidFM nanopipette. For the integration of biofunctional molecules into the adhesive-based patterns, a biotinylated phospholipid (biotin-PE) was admixed. The biotin–streptavidin complex [25] is widely used in biochemistry research, due to its strong binding affinity, which is commonly harnessed, e.g., in sensing applications [26].

#### 3.2.2. Patterning on Substrates with Different Wettability

Texture [27], surface chemistry [28], and wettability [29,30] are all known to be relevant parameters in the interactions of cells, platelets, and other species with biomaterials. Since surfaces can be chemically modified to achieve the desired biological response, being able to create adhesive patterns on differently terminated functional group surfaces is of high interest.

**Figure 3:**
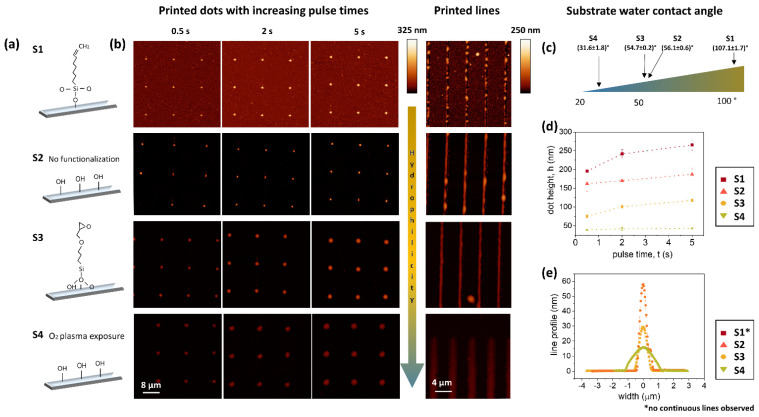
Influence of substrate wettability and chemistry on patterning. (**a**) Scheme of respective sample surface chemistry. (**b**) AFM images of polymer nanodots (left) and lines (right) patterned (right) on the functionalized substrates, showing increasing hydrophilicity from top to bottom (indicated by the arrow), written with the same working parameters. (**c**) Measured water contact angle for the different substrates. (**d**) Dot height on the different substrates as a function of the contact time. (**e**) Average line profiles showing different spreading behavior depending on the substrate functionalization.

To assess how the biofunctional inks behave when they are patterned on substrates of different surface chemistry, the biotinylated ink was used to write different features, while keeping the working parameters constant (Figure 3a,b).

For this, glass substrates were modified with different functional groups of self-assembled monolayers (SAMs). Briefly, the surfaces were functionalized by 7-octenyltrichlorosilane (S1), no treatment (S2), (3-glycidyloxypropyl)-trimethoxysilan (S3), and O_2_-plasma activation (S4), inducing hydrophobic (S1), medium hydrophilic (S2,S3), and strongly hydrophilic (S4) properties, respectively. The substrates were used two days after preparation, and the water contact angle was measured immediately before patterning (Figure 3c and Figure S2). To achieve comparable patterning, the same working conditions were employed. Nanodots were written, setting the contact time to 0.5, 2, and 5 s, subsequently, touching the substrate with 20 nN of force, and applying no pressure to the reservoir. Nanodots were successfully printed on all the substrates, and remarkably different heights were obtained, depending on the substrate treatment, while changes in the pulse time for ink dispensing had much less influence on the obtained heights (Figure 3d). The strongly hydrophobic 7-octenyltrichlorosilane-coated substrate (S1) leads to very high (over 200 nm) and confined features, while, on plasma-activated glass (S4), the dots considerably spread, causing much lower feature heights (around 40 nm). The differences between the outcomes of the patterns in S2 and S4 were surprisingly large, considering that they possessed basically the same surface chemistry, which reveals the importance of the substrate wettability (here, tuned by the oxygen plasma) in the spreading of the adhesive ink. The nanodot volume was quantified using the WSxM [24] flooding tool to prove that there were no significant differences in the amount of ink dispensed onto the different substrates (Figure S3). When considering the height change with pulse time, a clear increase in height with longer pulse time was only visible on the more hydrophobic substrates, but, even for these, the trend leveled off with longer pulse times.

The writing lines on the different SAMs turned out to be more critical, as can be inferred from Figure 3b,e. In the case of S1, the presence of the hydrophobic hydrocarbon chains compromised the stability of the features, which dewet and broke into droplets immediately after being printed. The rest of the line patterns (S2–S4), however, showed similar behaviour to that observed for the dots, where the highest lines were obtained on S2, while the spreading behaviour on the plasma-activated sample (S4) resulted in very low height features. The average values have been gathered in Table 1.

#### 3.2.3. Comparison of Mechanical Properties

**Figure 4:**
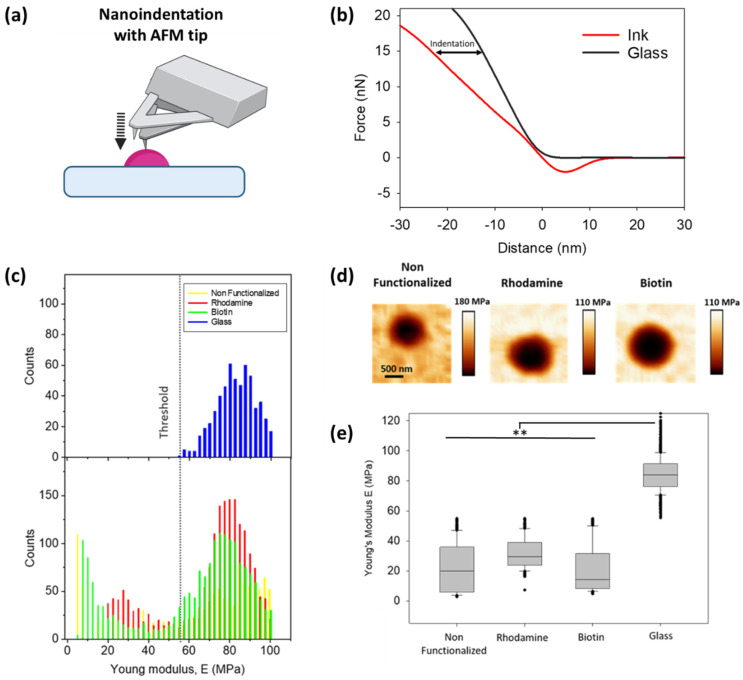
Influence of functional admixing on the mechanical properties of printed features determined by AFM nanoindentation. (**a**) Schematic of the nanoindentation experiments. (**b**) Typical AFM force-distance curve demonstrates the indentation on hard glass (black) to a compliant ink surface (red). (**c**) Quantification of Young’s modulus of bare glass and samples with modified inks. (**d**) Indentation map for the different composition nanodots (scale bar equals 500 nm for all images). (**e**) The Young’s modulus of modified nanodots shows small variation. Statistically significant difference determined by one-way ANOVA using Dunn’s test ** (*p* < 0.05).

It has often been shown that the mechanical properties of a substrate influence cell behaviour [31]. Hence, we assessed the influence of the biofunctionalized adhesive on the mechanical properties of the printed structures. For this, nanoindentation measurements over the dot features, from both non-functionalized and functionalized adhesive, were performed (Figure 4a). The force–distance curves extracted from these measurements were then analysed with JPK software, where the Young’s modulus was obtained after fitting the curves with a Hertz model (Figure 4b). The E-modulus values obtained on the functionalized and non-functionalized adhesive, together with the values on the glass surface, are presented as histograms in Figure 4c. Two peaks of distribution can be observed for all the ink samples. The measurement on the plain glass acts as a control, to differentiate the values taken on the dot features from those of the surrounding glass. The indentation maps show low E-modulus on the polymer structures (Figure 4d, dark area), while the surrounding glass exhibits a much higher value (Figure 4d, bright area). The distribution of the E-modulus values in the form of a box plot (Figure 4e) for the control glass and the inks (after subtracting the values from the glass) shows that on adding the functionality bearing phospholipids, the mechanical properties of the resulting printed features were not significantly altered. Although the addition of rhodamine or biotinylated lipids yields slightly stiffer materials, the obtained values stayed within the expected statistical variations, displayed as error bars. Additional measurements of Young’s modulus for different unfunctionalized patterns are given in the Supporting Information (Figure S1).

### 3.3. Biofunctionalization/Protein Binding

**Figure 5:**
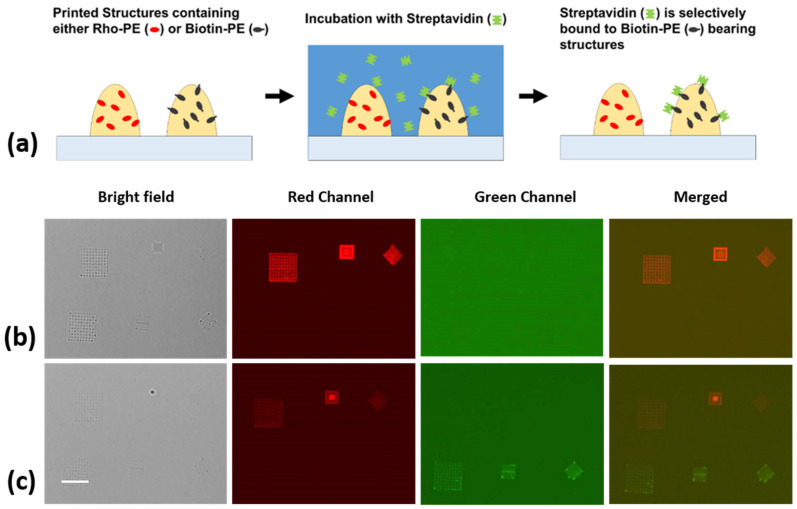
Biofunctionalization of adhesive-based structures by a model protein. (**a**) Scheme showing functionalization of the adhesive ink. Microscopy images of the (**b**) adhesive modified with rhodamine-PE (first row) and biotinylated adhesive structures (second row), and (**c**) the same adhesive structures after incubation with fluorescently labelled streptavidin, showing selective binding. Scale bar equals 40 µm for all images.

To prove the accessibility of the biotin moieties carried by the admixed phospholipids, the selective binding of a model protein was demonstrated (Figure 5a). For this purpose, new samples were prepared, where two functionalized adhesive-based inks were multiplexed onto the substrate, in the form of different geometrical micro-/nanostructures. In a preliminary stability experiment, conducted with non-cured samples, the written structures were incubated with streptavidin directly after patterning, with no further treatment, other than blocking the samples with BSA to avoid non-specific binding to the bare substrate areas. This experiment (Figure S4), showed highly specific streptavidin–biotin binding onto the functionalized structures, while the structures written with non-functionalized inks showed no sign of protein binding.

In a new, freshly made sample, the molecules were immobilized by curing the adhesive features under UV light for 5 min, to address the question of whether the biotin moieties stay on the surface and remain fully functional after curing. Working with fully cured samples can be critical for applications, as even when small-volume features are often already cured by exposure to ambient light, it might not be enough to fully cure thicker structures. The immersion of uncured adhesive in liquid might lead to rearrangement of the structures and the formation of droplets (Figure S4). Figure 5b,c displays the obtained fluorescent images before and after the incubation of fully cured patterns, with fluorescently labelled streptavidin. The emergence of the second row of patterns in the green channel after incubation constitutes an unequivocal sign of streptavidin binding to the biotin moieties.

## 4. Discussion and Conclusions

Three-dimensional nano and microprinting with UV-curable polymer inks has the potential to become a powerful and highly versatile method to produce bioactive and functional surfaces for biological research and biomedical applications. FluidFM is, in particular, suited for this kind of lithography, and widely available methacrylates, such as the commercial Loctite adhesive, can be employed [19].

We demonstrate that FluidFM allows the effective fabrication of micro-/nanostructures of different shapes. Importantly, the features of the printing structures can be adjusted by controlling the surface properties. By tuning the surface hydrophilicity with silanization or plasma cleaning, the height and width of the written structures can be modified. The highest structures were obtained on the most hydrophobic substrates, and the widest structures on the most hydrophilic substrates. This can be understood by the expected increasing contact angle of the ink with the substrate, which keeps the structure more confined, thus raising the height for the same volume of deposited ink for more hydrophobic inks. Our results can be used to purposely tune surface properties and to make informed choices for printing, when specific substrates are chosen for reasons of biological constraints.

However, it should be considered that being able to produce micro-/nanostructures in arbitrary shapes is only one aspect demanded in bioapplications. Most of the time, chemical or biochemical modifications are also key for obtaining the desired functionality. While a homogeneous chemical modification can easily be obtained in bulk by a post-functionalization step, targeted functionalization of specific micro-/nanostructures is hardly obtainable by conventional approaches. Towards this goal, we demonstrated the inherent functionalization of printed structures by admixing phospholipids to the base adhesive ink. In our approach, the resulting structures are directly functionalized without any need for further steps. Furthermore, this gives the opportunity for multiplexing, as structures can easily be written next to each other with different inks. Here, we demonstrated the concept by admixing a fluorescently labelled phospholipid or a biotinylated phospholipid, respectively. With simple conventional blocking agents, such as BSA, unspecific adhesion is blocked and streptavidin is highly selective, binding only to the structures made with the ink containing biotinylated phospholipid. This proves that the biotin moieties remain accessible at the interface of the structures to the liquid phase, probably preferentially oriented outwards of the polymer bulk by their amphiphilic natures (the hydrophilic head group is modified with the biotin moiety and hydrophobic hydrocarbon chains at the tail end). Remarkably, the biotin motif offers a wider range of possibilities for biofunctionalization, as it is one of the most widespread binding tags in biotechnology, with a myriad of compounds available with biotin modifications that could be bound over a streptavidin linker to such structures. In addition, other lipid modifications will likely work in a similar fashion, e.g., to introduce metal-chelating lipids, such as 1,2-dioleoyl-sn-glycero-3-[(*N*-(5-amino-1-carboxypentyl)iminodiacetic acid)succinyl] (nickel salt) (18:1 DGS-NTA(Ni)), to allow the binding of polyhistidine(His)-tagged proteins, further expanding the multiplexing capabilities by utilizing orthogonal binding tags. The admixing did not significantly alter the printing properties or mechanical properties of the printed structures; therefore, it is a straightforward process to include different functionalization and no interference with, e.g., changes in mechanical cues, as stiffness to the cells growing on the structures is to be expected.

Another important concern when considering bioapplications is the biocompatibility of the involved materials. While methacrylates are problematic in this regard, the toxicity of the material is mainly conveyed by residual monomers seeping out [32]. For the comparably thin structures produced in FluidFM printing processes, curing will generally be complete with not much monomer left, and, if needed, biocompatibility could be further increased by additional treatments, such as ethanol washing [33].

In summary, our results show the successful functionalization of polymer ink with model biomolecules, and provide proof of principle as to how biologically relevant species can be incorporated into the direct-patterned nanostructures, without significantly altering their global properties. Overall, these results demonstrate the potential for the direct printing of functionalized structures via FluidFM, or similar dispensing techniques, for the creation of bioactive, protein-presenting micro-/nanostructures for bioapplications.

## Figures and Tables

**Table 1:** Height of adhesive lines printed on differently functionalized substrates.

Substrate Label	S1	S2	S3	S4
Functionalization	7-octenyl trichlorosilane	None	(3-glycidyl oxypropyl)-trimethoxysilan	O_2_ plasma activation
Width * (nm)	- **	300 ± 21	524 ± 26	1457 ± 65
Height (nm)	- **	68 ± 16	32 ± 3	15 ± 1
Aspect ratio ***	-	0.23 ± 0.04	0.06 ± 0.01	0.01 ± 0.01

* full width at half maximum (FWHM). ** no continuous line writing was obtained. *** calculated as height/width, error calculated by error propagation.

## Data Availability

The data presented in this study are available on reasonable request from the corresponding authors.

## Supplementary Materials

The following are available online at https://www.mdpi.com/article/10.3390/polym14071327/s1: Figure S1: indentation maps of different structures; Figure S2: water contact angles on the different surface functionalization; Figure S3: calculated dot volumes on substrates with different functionalization; Figure S4: additional incubation experiments.
