# Supplementary material for: Integration of Biofunctional Molecules into 3D-Printed Polymeric Micro-/Nanostructures
Source: Polymers (Basel). 2022 Mar 25;14(7):1327. doi: 10.3390/polym14071327 (PMC9002480; doi:10.3390/polym14071327)
Supplement: Supplementary file 1 [file polymers-14-01327-s001.zip › polymers-1649443-supplementary(1).pdf]

polymers

MDPI

Supporting Information for

Integration of Biofunctional Molecules into 3D-printed Polymeric Micro-/Nanostructures

Eider Berganza [1†], Gurunath Apte [1,2†], Srivatsan K. Vasantham [1], Thi-Huong Nguyen [2,3*] and Michael Hirtz [1,*]

1 Institute of Nanotechnology (INT) and Karlsruhe Nano Micro Facility (KNMF), Karlsruhe Institute of Technology, 76131 Karlsruhe, Germany
2 Institute for Bioprocessing and Analytical Measurement Techniques (iba), 37308 Heilbad Heiligenstadt, Germany
3 Faculty of Mathematics and Natural Sciences, Technische Universität Ilmenau, 98694 Ilmenau, Germany
* Correspondence: thi-huong.nguyen@iba-heiligenstadt.de (T.H.N.); michael.hirtz@kit.edu (M.H.)
† These authors contributed equally to the work

S1. Indentation Maps of different Structures

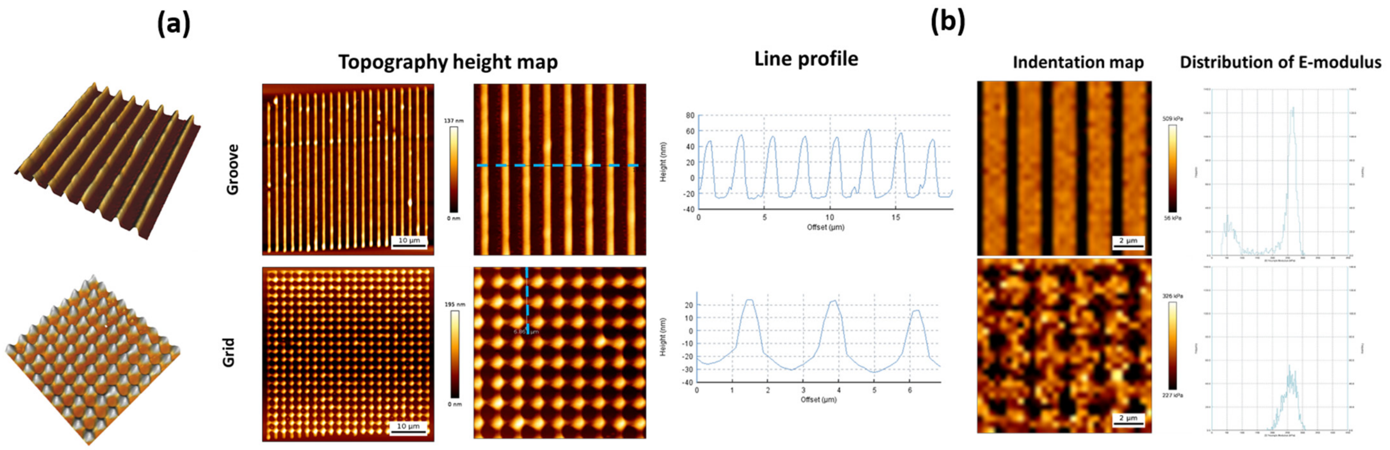

Figure S1. Indentation maps of different structures. **(a)** Atomic Force Microscopy topography images of different printed polymer structures. **(b)** Corresponding indentation maps and calculated distribution of Young's modulus of the lines and grid structures.

S2. Dot Volume Comparison on Surface with different Wettability

To validate the AFM measurements of the adhesive nanodots printed on differently functionalized surfaces, the nanodot volumes were calculated using the flooding tool WSxM software. As can be seen, the volumes of all dots printed under the same writing conditions have comparable values, regardless of the characteristics of the substrate on which they were printed.

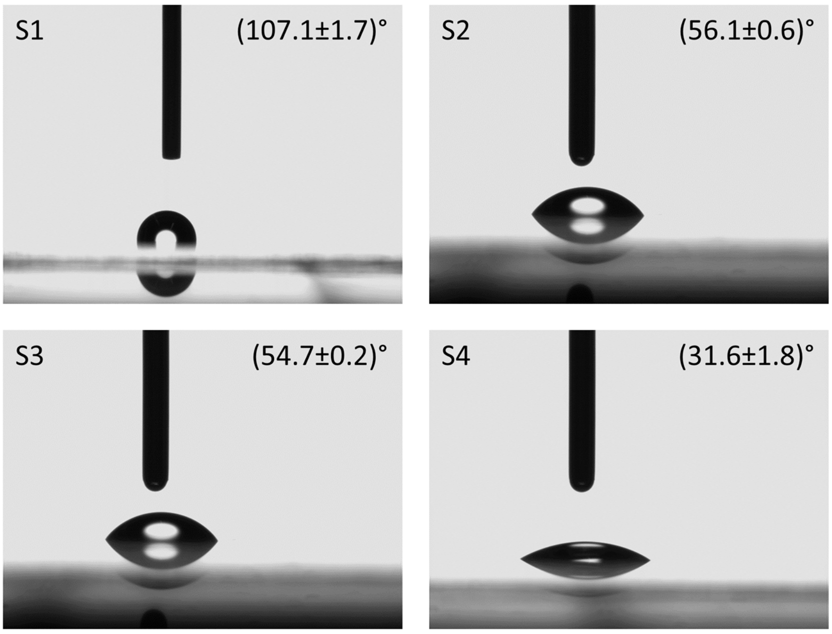

Figure S2. Water contact angles on the different surface functionalizations. 7-octenyltrichlorosilane (S1), none (S2), (3-glycidyloxypropyl)-trimethoxysilan (S3), and O_2 plasma-activated (S4), all measured 2 days after preparation, immediately before use in the printing experiments.

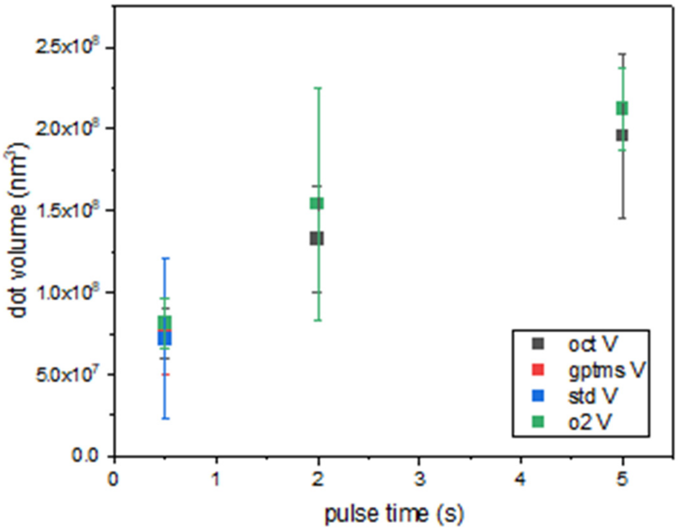

Figure S3. Calculated dot volumes on substrates with different functionalization.

S3. Additional Incubation Experiments

Biotin-functionalized adhesive structures were prepared in different shapes. The full-square in the left (Figure S3a), presents a larger thickness. Similar to the experiments reported in the main manuscript, BSA blocking and streptavidin binding steps were performed on this non-cured sample shown here and the steps were monitored through fluorescent microscopy. As we can see from the images, immersion in liquid results in rearrangement of the adhesive and phospholipid mixture, which can compromise the integrity of the patterned structures.

In Figure 3b, negative controls are shown, proving that the streptavidin binds solely to biotin functionalized adhesive and not to the pure adhesive nanostructures.

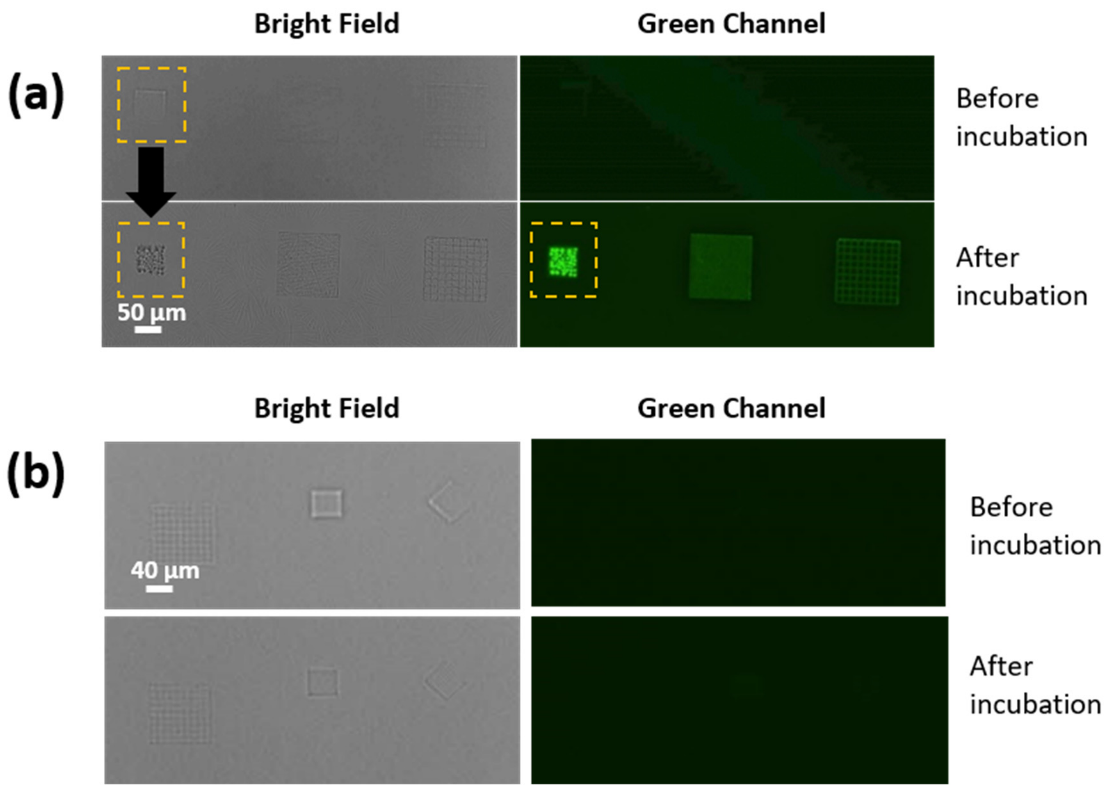

Figure S4. Additional incubation experiments. Bright-field and FITC channel images of as-prepared and incubated structures of **(a)** non-cured biotinylated adhesive and **(b)** cured non-functionalized adhesive, showing no unspecific binding fluorescent signal.
